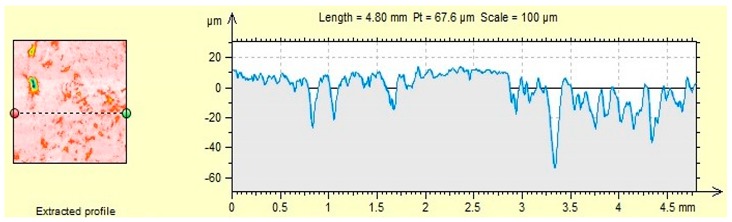# Physical Properties of Mineral and Recycled Aggregates Used to Mineral-Asphalt Mixtures

**DOI:** 10.3390/ma12203437

**Published:** 2019-10-21

**Authors:** Wojciech Andrzejuk, Danuta Barnat-Hunek, Jacek Góra

**Affiliations:** 1 Faculty of Technical Sciences, Pope John Paul II State School of Higher Education in Biała Podlaska, Sidorska St. 95/97, 21-500 Biała Podlaska, Poland; 2 Faculty of Civil Engineering and Architecture, Lublin University of Technology, Nadbystrzycka St. 40, 20-618 Lublin, Poland; d.barnat-hunek@pollub.pl (D.B.-H.); j.gora@pollub.pl (J.G.)

**Keywords:** aggregate from sanitary ceramic wastes, granodiorite, dolomite, microroughness, microstructure

## Abstract

This article presents test results and examines the possibilities of using aggregate from ceramic waste for mineral-asphalt mixtures. In addition, the mineral composition, physical and mechanical properties of aggregates from natural raw materials such as dolomite, granodiorite and waste ceramic aggregate (introduced as a partial substitute for the main aggregate) were analyzed. The shape of grains was examined by determining the shape and flatness index of aggregates, resistance to grinding and frost resistance. The tested properties have a direct impact on the durability of road surfaces. To this end, the adhesion of asphalt to the surface of the aggregates used was additionally determined. Determination of surface roughness and two-dimensional (2D) topography of tested aggregates was carried out. The aggregates microstructure examination, coupled with the energy-dispersive X-ray spectroscopy (EDS) analysis, was conducted to determine the morphology and texture of the aggregates as well as to identify the basic chemical components.

## 1. Introduction

Aggregates from natural raw materials have been widely used for wear layers of asphalt surfaces. Grits from igneous, sedimentary and metamorphic rocks are most commonly used, and the use is often of a regional nature and depends on the availability of a given type of aggregate. In Poland, basalts, granites and granodiorites, dolomites, limestones or quartzite sandstones are often used.

When considering the mineral-asphalt mixture (MAM) as a two-component composite, its properties will be derived from the parameters of both asphalt and aggregate. The choice of aggregate is primarily determined by its physical and mechanical properties. Basic properties of aggregates include mineralogical composition, surface texture and grain shape, dustiness, porosity, frost resistance, resistance to abrasion and polishing, and asphalt absorption capacity [1,2,3,4,5].

When choosing an aggregate for an asphalt mixture, one should be aware of not only the immediate effects of its interaction with asphalt, which are characterized by specific physical and mechanical properties, but also of the effects resulting from prolonged contact of asphalt with aggregate, which may include loss of extracted content from MAM soluble asphalt. Phenomena occurring in the contact zone between the binder and the aggregate surface play an important role in shaping the properties of the asphalt mixture and the construction layer made of it [6].

Washability is an indirect measure of the strength of the bond between the tested binder and various aggregates or various aggregates with a given asphalt. Adhesion of the binder to the aggregate is the force needed to tear off the asphalt layer from the stone material. Adhesion is influenced by many factors, the most important of which are: the chemical composition of asphalt and the mineralogical composition of the aggregate as well as the shape and nature of the aggregate grain surface. To obtain a guarantee of permanent binding of the binder to the aggregate in the presence of water, the wetting capacity of the aggregate surface by asphalt should be greater than the wetting capacity by water. Therefore, in order to improve the adhesion of asphalt to the aggregate, adhesive additives are used [7].

Asphalts, due to their affinity, have better adhesion to alkaline rocks than to acidic ones. The less silica is in the rock from which the aggregate originates, the better is the adhesion. The use of alkaline aggregates—characterized by high energy potential—in mineral-asphalt mixtures, ensures a durable and strong bond between asphalt and aggregate. For this reason, these aggregates are safely used in asphalt surfaces [8].

Physico-chemical factors affecting the adhesion of asphalt to aggregate include:

- The chemical nature of the aggregate; depending on the amount of silica contained in the aggregate, we divide them into acid, alkaline and intermediate,
- Physicochemical properties of asphalts—such as viscosity and adhesion, which depends on the content of acidic compounds.

Types of aggregates obtained from various types of rocks and the amount of silica contained in them are presented in [9]. Depending on the silica content, aggregates are divided into [9]:

- Acid—with a silica content >65%,
- Alkaline—with a silica content <55%,
- Intermediate—with a silica content in the range 55% to 65%.

Most aggregate surfaces are not electrically neutral [2]. Silica, which is the main component of igneous rocks, has a weak negative charge, which is the result of the presence of not completely electrically neutral oxygen atoms on the aggregate surface [10]. The chemical adhesion of the asphalt film to the aggregate surface is the result of the interaction of relatively weak, dispersed electric charges [10]. Acid aggregates have stronger hydrophilic properties than alkaline aggregates [11]. Asphalt, which is an electronegative colloid, shows better adhesion to alkaline, electropositive aggregates than to acid aggregates [12]. Extensive research on the adhesion of asphalt to aggregates was carried out in the USA [13]. Various types of aggregates were tested, including: limestone, porphyry, granite, dolomite and others. Based on the obtained research results, it was found that dolomite aggregate has good adhesion (approximately 90%), and porphyry—poor adhesion (about 30%) [13]. The suitability of high-quality dolomite aggregates for asphalt mixtures has also been confirmed in other studies [14].

The degradation of the natural environment observed in the recent years, resulting from a rapid civilizational development has led to the fact that alternative raw materials using recycled materials are being sought. Examples of waste materials used in mineral asphalt mixtures are fly ash and waste engine oil [15,16].

This article presents the results of research and examines the possibilities of using aggregate from ceramic waste for mineral-asphalt mixtures. So far, this type of aggregate has been tested for applications in cement concrete. It has been shown that concrete with this type of aggregate is characterized by high strength and frost resistance, high abrasion resistance and is resistant to high temperatures [17,18,19,20]. Despite the fact that ceramic sanitary products are very durable, more and more often these products are replaced not after technical defect but for aesthetic reasons, for example. This practice results, on one hand, in the increased production of these products, and, on the other hand, in large quantities of this waste appearing at landfill sites. For these reasons innovative solutions for their recycling are being sought.

The few studies presented confirm the possibility of using a ceramic waste aggregate for asphalt mixtures as a partial substitute for natural aggregate [21,22]. Asphalt mixtures in which the limestone aggregate and an aggregate mixture containing 30% substitute in the form of ceramic waste aggregate were used. It has been established on the basis of research that waste ceramic aggregates can be used in such amounts in asphalt mixtures—however, only in the case of medium-low traffic. Additionally, based on the research by [22], it was found that asphalt mixtures with the addition of waste ceramic aggregates can meet the requirements for surface performance. It is recommended to add less than 40% waste ceramic aggregate to asphalt mixtures to replace natural coarse aggregates, taking into account its effect on the mixtures tested. The addition of these aggregates can reduce the thermal conductivity of asphalt mixtures, which in turn reduces the surface temperature gradient [22].

To the best of our knowledge, waste ceramic aggregate has not been tested in terms of microstructure, frost resistance, microroughness and its influence on the adhesion between asphalt and waste ceramic aggregate. Therefore, it was determined with a special for microroughness and frost resistance relationship.

In the presented article, the mineral composition, physical and mechanical properties of aggregates from natural raw materials (dolomite, granodiorite) and waste ceramic aggregate (introduced as a partial substitute for the main aggregate) were analyzed. The tested properties have a direct impact on the durability of road surfaces. To this end, the adhesion of asphalt to the surface of the aggregates used was additionally determined.

## 2. Materials and Methods

### 2.1. Material Properties

**Figure 1:**
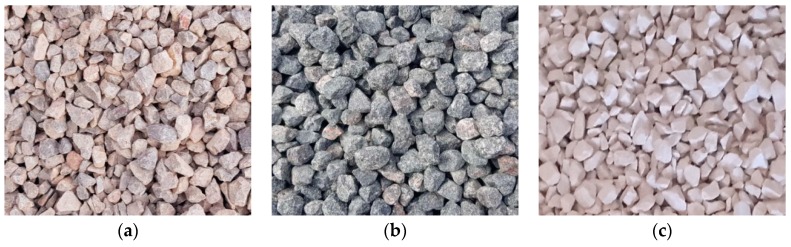
Tested aggregates: (**a**) dolomite 8/11.2, (**b**) granodiorite 8/11.2, (**c**) aggregate obtained from sanitary ceramics 4/8.

Three types of aggregate were used for the study: dolomite (Figure 1a), granodiorite (Figure 1b) and recycled ceramic waste aggregate (Figure 1c), which was obtained from a waste heap at an industrial plant producing sanitary products. It was primarily a sanitary assortment with cracks, damaged enamel or an uneven surface. The waste obtained in this way was subjected to a crushing process in jaw crushers.

Dolomite and granodiorite are among the aggregates traditionally used in mineral-asphalt mixtures. On the basis of earlier studies [23], it was assumed that 20% to 30% of the addition of ceramic waste aggregate can be used as a substitute for aggregates traditionally used in mineral-asphalt mixtures used for construction of road surfaces. The granodiorite aggregate used comes from Tomashgorod Rokytne district, Rivne—a region in Ukraine, and dolomite aggregate from the Polish Dolomite Mine Piskrzyń. Granodiorite and dolomite aggregate with grain size d ≥ 8 mm and D ≤ 16 mm were used in the tests. The properties of dolomite and granodiorite aggregate are shown in Table 1.

Two types of asphalt were used in the affinity tests between aggregate and asphalt: 50/70 road asphalt and 45/80-55 polymer modified asphalt. The parameters of these binders are listed in Table 2 and Table 3.

### 2.2. Methods

The first test was to determine the shape of the grains. The determination consists in determining the *SI* shape index calculated as a percentage of the mass of irregular grains in the aggregate with a ratio of their length *L* to the thickness *E* greater than 3, separated from the sample as a result of grain measurements using a linear measurement device. The test was carried out according to PN-EN 933-4 [25].

Another test was determining the aggregate flatness index *FI* according to PN-EN 933-3 [26]. The study was carried out in two stages. To begin with, the sample was separated into individual d_i_/D_i_ fractions, and then these fractions were sieved on finger screens.

Subsequently, aggregate resistance to grinding was determined using the Los Angeles method according to PN-EN 1097-2 [27]. The purpose of this test is to recreate the operating conditions of aggregate in road surface and assess its resistance to abrasion and disintegration.

The next test carried out was aggregate frost resistance testing according to PN-EN 1367-6 [28]. The method chosen was to determine the loss of aggregate grain masses as a result of a specific number of freezing cycles impacting on the aggregate and thawing of the moistened aggregate sample, which was expressed as a percentage.

For the purpose of determining the adhesion of asphalt binder to the aggregate, an affinity test between aggregate and asphalt binder was carried out using the rotating bottle method according to PN-EN 12697-11, method A [29]. In this method, the affinity is determined on the basis of a visual assessment of the asphalt coverage of the non-concentrated asphalt-surrounded aggregate after it has been subjected to mechanical agitation in the presence of water.

Determination of surface roughness and 2D topography was performed on the T8000 RC120-400 JENOPTIC device, Jena, Germany, similarly to the procedure described by Barnat-Hunek et al. [30,31]. Measurements were carried out using a standard graphical user interface (GUI), enabling the calculation of all parameters of the considered roughness profiles, as well as an assessment of the geometrical features of aggregates. The device has a resolution of 50 nm, the wavecontour™ digital measuring system with digital linear scales in the Z and X axes. Roughness was measured on inclined or bent surfaces with a resolution of 6 nm in the measuring range of 4.8 mm. Five randomly selected fragments of aggregate samples were tested by measuring surface porosity 4.5 × 4.5 mm^2^. Images of the surface structure and profilograms obtained directly using a 3D profilographometer reflected the obtained roughness parameters characterizing the surface of the tested aggregates.

The aggregate surface can be characterized by the following parameters [30,32]:

R_a_—Average Roughness defined as the average deviation of the profile in relation to its mean line and parameter more sensitive to peaks and valleys;

R_p_—Maximum Peak Height as the maximum height of peak within evaluation length;

R_v_—Maximum Valley Depth as the maximum depth observed within the evaluation length;

R_max_—Maximum Peak-to-Valley Height understood as the maximum peak-to-valley height within any of the sampling lengths; R_max_ = R_v_ + R_p_.

SEM (Quanta FEG 250 microscope by FEI, Hillsboro, OR, USA), equipped with a system for the chemical composition analysis based on the energy-dispersive X-ray spectroscopy (EDS) manufactured by EDAX (Mahwah, NJ, USA), was used for determining the morphology and porous structure of aggregates.

## 3. Results

### 3.1. Physical and Mechanical Characteristics of the Tested Aggregates

The results of determining the SI shape index, the flatness index FI, the resistance to grinding and frost resistance for individual aggregates are presented in Table 4.

The results of the affinity determination between the aggregates analyzed and 50/70 road asphalt and 45/80-55 polymer modified asphalt are summarized in Table 5.

If in the result of cooking the test result is lower than 80%, an adhesive should be added to the mineral-asphalt mixture. The average adhesion of Polish oxidized asphalts to various types of aggregates, determined by the cooking method, normally amounts to [9]:

- 30% to 90% for basalt;
- 60% to 90% for limestone;
- 40% to 80% for granite.

### 3.2. Microroughness

The characteristics of microroughness obtained for the tested aggregates shown in Table 6.

### 3.3. Microstructure of Aggregates

**Figure 2:**
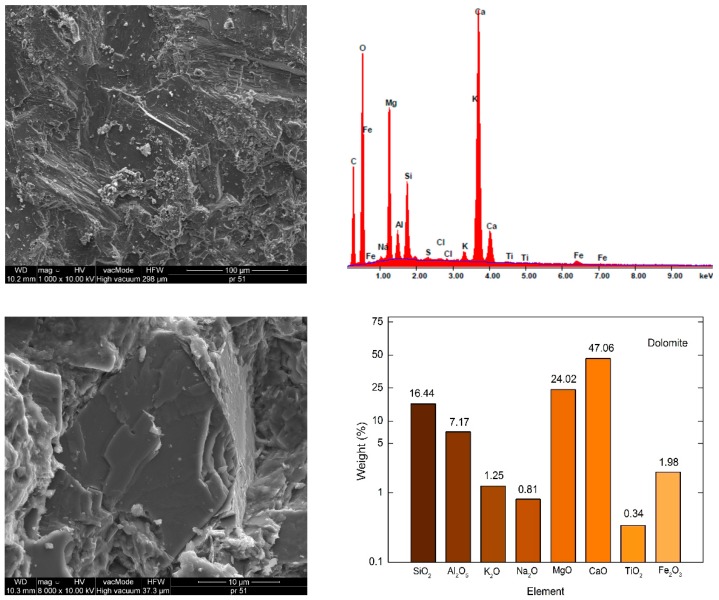
Dolomite SEM microstructure (1000× and 8000×) and elemental analysis results in the EDS micro area.

**Figure 3:**
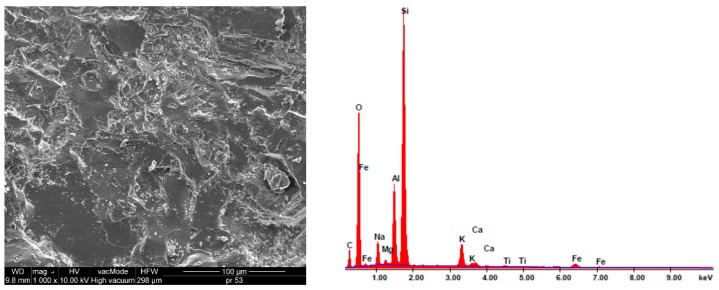

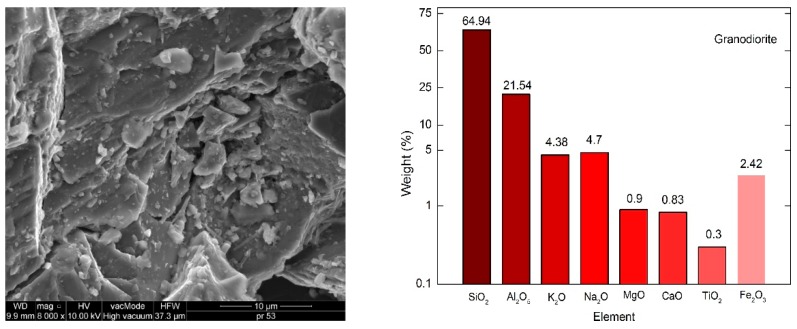
Granodiorite SEM microstructure (1000× and 8000×) and elemental analysis results in the EDS micro area.

**Figure 4:**
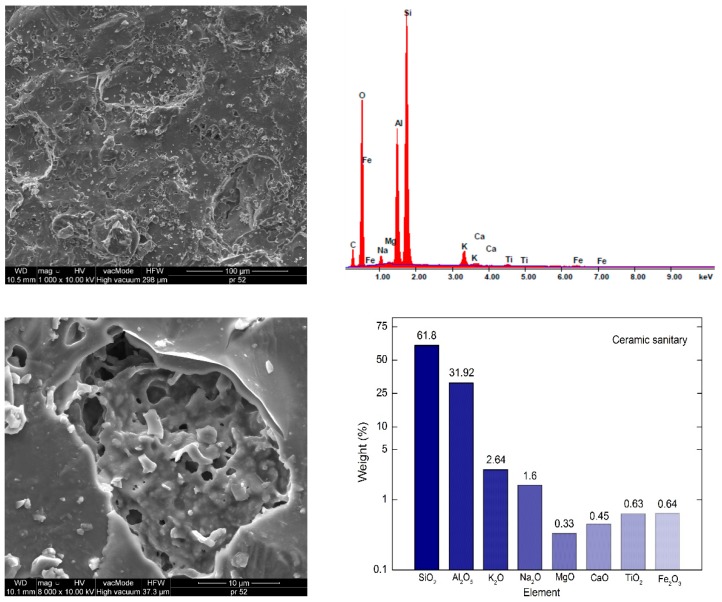
Sanitary ceramics SEM microstructure (1000× and 8000×) and elemental analysis results in the EDS micro area.

Figure 2, Figure 3 and Figure 4 present the SEM images showing the microstructure of aggregates. The chemical composition of the aggregates is presented based on energy dispersive spectrometry (EDS).

## 4. Discussion

### 4.1. Physical and Mechanical Characteristics of the Tested Aggregates

According to Polish technical requirements [33] regarding aggregates used for the asphalt concrete wear layer, the shape index SI and the flatness index FI for coarse aggregate should not be higher than SI_25_ and FI_25_ for traffic load TL1-TL2 and SI_20_ and FI_20_ for traffic load TL3-TL6. Therefore, the tested aggregates meet the requirements of technical specification presented above for all categories of traffic (Table 4). The shape index and flatness index are related to the resistance of asphalt surface to permanent deformations, which was also described in [14]. It is recommended to use aggregates with shape indexes SI_20_ and FI_20_. The increased presence of spherical aggregate grains can cause a decrease in internal friction in the mixture, and thus a decrease in shear strength. This in turn translates into increased susceptibility to plastic deformation of the road surface.

Aggregate in road surface is subjected to abrasion and grinding as a result of car traffic and internal friction of aggregate against each other. The wear layer is particularly exposed to these factors. Too much susceptibility of aggregate to abrasion and grinding causes faster surface wear [34]. It is similar with aggregate frost resistance, especially for wearing layers, which often have direct contact with very harsh climatic conditions, as well as aggressive substances used as part of winter maintenance works. Insufficient aggregate frost resistance will lead to faster degradation of asphalt road surface. All tested aggregates met the requirements of Polish technical specification [33] regarding LA grinding resistance for all traffic loads (Table 4). According to the Polish technical specification, the resistance to crushing LA of coarse aggregate of 10/14 to asphalt concrete wear layer should not be higher than LA_30_ for traffic load TL1–TL4 and LA_25_ for traffic load TL5–TL6. The lowest Los Angeles index with a value (16%) was obtained by granodiorite aggregate and the highest by dolomite aggregate (23%). The results regarding frost resistance of tested aggregates are similar (Table 4). All analyzed aggregates met the requirements of WT-1, which assume that the frost resistance category of coarse aggregate for asphalt concrete wear layer should not be higher than F_NaCl_10 for traffic load TL1–TL2 and F_NaCl_7 for traffic load TL3–TL6. Ceramic aggregate obtained the best results in frost resistance tests. Waste ceramic aggregate was characterized by the smallest percentage of mass loss (1.2%) and dolomite aggregate had the largest percentage (6.3%).

To the best of our knowledge, waste ceramic aggregate has not been tested in terms of the criterion of the Polish technical requirements for the application of road pavements.

When analyzing the results of the test for resistance to grinding and frost resistance, it can be observed that in the case of the tested waste ceramic aggregate, higher resistance to grinding also determines higher frost resistance, and both of these properties of the aggregate used for the mineral-asphalt mixture have a significant impact on the wear and durability of the road surface [2].

### 4.2. Microroughness

The presented roughness test showed a variation in the geometrical structure of the aggregate surface, which is related to the mechanical adhesion of asphalt to the aggregate (Table 6). The rough aggregate surface may be susceptible to abrasion, grinding, and should have a physicochemical affinity ensuring adequate adhesion of the asphalt binder to the aggregate.

Sanitary ceramics is characterized by the highest average roughness (5.61 µm), while the lowest one can be observed for dolomite (2.06 µm). However, R_a_ is only the average roughness value, which does not indicate the largest deviations on the surface of the material, therefore in the following part of the article R_max_ values were analyzed, which indicate the highest amplitude constituting the sum of the largest peak and the deepest valley (R_p_ + R_v_).

The analyzed roughness parameters indicate that the average roughness R_a_, the maximum peak height R_p_ and the maximum valley depth R_v_ depend on the type of aggregate. R_max_ is the highest for sanitary ceramic aggregate and it is higher than by 61.5% R_max_ dolomite. The difference between sanitary ceramics and granodiorite is much lower and amounts to 19.4%.

The high roughness of the ceramic aggregates and granodiorite caused a high frost resistance of the aggregate (Table 4) and the smallest weight loss (1.2% and 1.8%, respectively) among the analyzed aggregates after freezing-thawing cycles in the presence of chlorides. The process of creating ice crystals, including ice in the presence of chlorides, is possible in numerous pores and empty spaces on the surface of the material. Therefore, there is no significant damage to the surface or mass loss of the aggregate.

**Figure 5:**
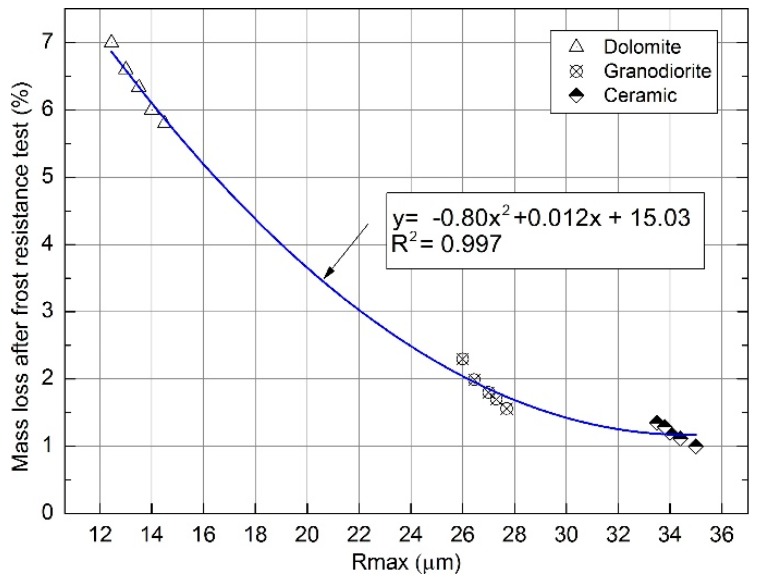
Correlation between roughness Rmax and average mass loss after aggregates resistance test.

A parabolic relationship between roughness, more precisely the maximum peak-to-valley height R_max_, and the average mass loss of the samples after the frost resistance test was observed. A regression model with two variables was used (Figure 5). The correlations described by the formula $y=-0.799x^{2}+\;0.012x+15.031$ indicate a good coefficient of determination R^2^ = 0.997.

These correlations mainly result from the absorbability and wettability of aggregates, the results of which were presented by [23,35]. This is because water absorption is related to the contact angle as well as the roughness.

Dolomite–aggregate with the smallest roughness, the smallest maximum valley depth R_v_ and R_max_, shows the highest affinity with asphalt (60% to 70% after 6 h, depending on the asphalt). According to Hay et al. [36] the penetration of liquids (in this case asphalt) is hindered by many pores and empty spaces on the surface of the material, except for materials with high roughness. This is particularly evident when using high-viscosity liquids, which settle on the peaks found on a rough surface [36]. Representative 2D surface profilograms (Table 6) of aggregates show visible differences in their micro roughness. The obtained results regarding the surface roughness parameters of the aggregates considered are reflected in the results of the asphalt and aggregate affinity. The adhesion to aggregate decreases when air bubbles are trapped under the asphalt layer. Most likely, when the roughness is too high, the adhesion between asphalt and aggregate decreases—as is the case with granodiorite—which has the lowest affinity among the aggregates analyzed (Table 5) and high roughness. Roughness is also associated with porosity of the material, which affects the adhesion, as mentioned by Sadowski Ł. et al. [7].

### 4.3. Microstructure of Aggregates

The aggregate microstructure in scanning microscopy images is shown in Figure 2, Figure 3 and Figure 4. The dolomite structure is very compact, there are no visible pores, scratches nor cracks. EDS analysis showed that calcium oxide (47.06%) and magnesium oxide (24.02%) predominate in the composition of dolomite. The SiO_2_ content is 16.44%, which qualifies dolomite to alkaline aggregates [9]. The structure of granodiorite and sanitary ceramics is somewhat different, free spaces are visible, and in the case of granodiorite -cracking (Figure 3). Pores exceeding 5 μm in diameter were observed in the ceramic aggregate (Figure 4). A large number of pores increases the absorbability of ceramics compared to granodiorite and dolomite [23,35].

The dominant component in both aggregates is silica, the content of which exceeds 61% and Al_2_O_5_-21.5% in the case of granodiorite and 31.9% in the case of sanitary ceramics, respectively (Figure 3 and Figure 4). Therefore, according to the literature, these aggregates can be considered acid aggregates [8,9]. In sanitary ceramics, much lower (3.5–4 times) Fe_2_O_3_ content, compared to other analyzed aggregates, was observed.

Naga et al. showed in their research a similar composition of granodiorite to the one analyzed in the paper [37]. The amount of silica in the examined granodiorite was 69%, and Al_2_O_3_ 27.29%. The research of Naga et al. showed that granodiorite is a medium to coarse grained material with hypidiomorphic granular texture. Sericite and kaolinite occur as secondary minerals with additional minerals such as apatite, zirconium and iron oxides. Quartz crystals are the second most abundant mineral and occur as subhedral to anhedral crystals [37].

The results of tests on the chemical composition of aggregates are reflected in the tests on the affinity of asphalt to aggregates. This affinity is the highest in the case of alkaline rock–dolomite and amounts 60% to 70% (Table 5). The weakest, unsatisfactory affinity was obtained in the case of the aggregate with the highest silica content—granodiorite. Artificial aggregate showed a slightly higher affinity with asphalt.

Ovidijus Šernas et al. [14] showed in their research that the affinity of asphalt modified with PMB 45/80-55 polymer was higher for dolomite (85%) than for granite (80%). In our research, these values are much lower. When granodiorite and sanitary ceramics are used in asphalt mixtures, in order to improve the adhesion of aggregate and asphalt, adhesive agents in the form of admixtures should be used [9,38,39].

## 5. Conclusions

The results of the tests showed that the tested aggregates have sufficient properties regarding grain shape, frost resistance and resistance to grinding in terms of their application to the wear layer of asphalt concrete surface. They also noted the adhesion of asphalt to the aggregate grains. Insufficient adhesion of asphalt to the aggregate leads to faster wear of the surface. The tests carried out based on the Polish technical requirements have shown that:

- Crushed waste ceramic aggregate meets the requirements for the shape of the aggregate grains, and at the same time contains a larger but acceptable amount of irregular grains than granodiorite and dolomite aggregate.
- All aggregates have sufficient resistance to cyclic freezing and thawing as well as resistance to grinding. Resistance to grinding is a particularly important feature in the case of aggregates used for wearing layers, where, as a result of car traffic, abrasion and grinding of aggregate grains occurs. It was noticeable that the higher resistance to aggregate grinding also influenced its higher frost resistance, which in turn is of great importance in terms of wear and durability of asphalt surface.
- In the case of tested aggregates and applied binders, the adhesion of asphalt binder to aggregate grains is not sufficient. Adhesives that improve the adhesion of asphalt to the aggregate should be used—which in turn will ensure permanent bonding of its grains. Subsequently, it will lead to the possibility of designing and making asphalt surfaces with high durability and good operational properties.

Another direction of research of the authors will be research with the use of selected adhesion agents, which will improve the adhesion of asphalt to aggregate—especially ceramic aggregate.

## Figures and Tables

**Table 1:** Properties of granodiorite and dolomite aggregates [24].

	Granodiorite	Dolomite
Specific density (kg/dm^3^)	2.67	2.80
Bulk density (kg/dm^3^)	2.63	2.60
Absorptivity (WA_24_), %	WA_24_2	WA_24_2
Abrasion resistance (MDE), %	M2	M_DE_10
Polished stone value (PSV)	PSV_50_	PSV_44_
Frost resistance (F), %	F_1_	F_2_

**Table 2:** Parameters of road asphalt binder 50/70.

Parameter	Unit	Value
Penetration at 25 °C	1/10 mm	50–70
Softening point	°C	46–54
Embrittlement temperature	°C	≤−8
Ignition temperature	°C	≥230
Solubility	% m/m	≥99.0
Mass change (absolute value)	% m/m	≤0.5
Remaining penetration at 25°C	%	≥50
Softening point increase	°C	≤9

**Table 3:** Parameters of polymer modified asphalt binder 45/80-55.

Parameter	Unit	Value
Penetration at 25 °C	1/10 mm	45–80
Softening point	°C	≥55
Tensile force (strain energy)	J/cm^2^	≥3 at 5 °C
Weight change after aging	% m/m	≤0.5
Remaining penetration at 25 °C after aging	%	≥60
Increase in softening temperature after aging	°C	≤8
Ignition temperature	°C	≥235
Embrittlement temperature	°C	≤−15
Elastic recovery at 25 °C	%	≥70
Storage stability—softening difference	°C	≤5
Softening temperature decrease after aging	°C	TBR
Elastic recovery at 25 °C after aging	%	≥50

**Table 4:** Grain shape determination results—SI shape index.

Properties		Granodiorite	Dolomite	Ceramic Aggregate
Shape index SI (%)		0	4	20
Flatness index FI (%)		1	6	16
LA grinding resistance, %		16	23	22
Frost resistance	Freezing–thawing; percentage mass loss	1.8	6.3	1.2
	F_NaCl_ category	F_NaCl_5	F_NaCl_7	F_NaCl_5

**Table 5:** Results of the affinity determination between the aggregates and asphalt binder used.

Properties	Granodiorite		Dolomite		Ceramic Aggregate	
	6 h	24 h	6 h	24 h	6 h	24 h
Road asphalt 50/70	40	30	70	45	60	50
45/80-55 polymer modified asphalt	40	20	60	40	50	40

**Table 6:** Microroughness characteristics and representative 2D profilograms of aggregates.

	R_a_ (µm)	R_p_ (µm)	R_v_ (µm)	R_max_ (µm)
(a) Dolomite	2.06	4.80	8.29	13.09
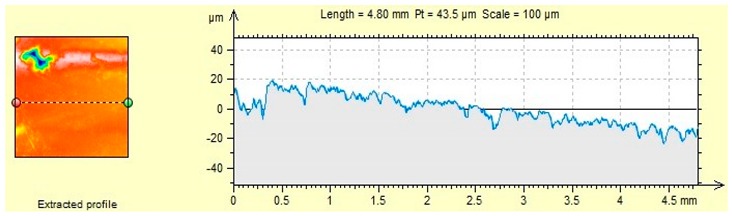				
(b) Granodiorite	4.43	11.3	16.1	27.4
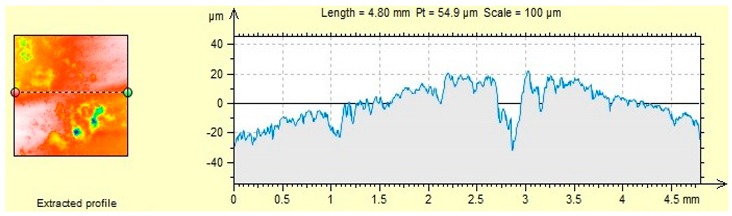				
(c) Ceramic sanitary	5.61	11.4	22.6	34.0